# Iodido[1-(propan-2-yl­idene)thio­semi­carbazide-κ*S*]bis­(triphenyl­phosphane-κ*P*)copper(I)

**DOI:** 10.1107/S1600536812044066

**Published:** 2012-10-31

**Authors:** Yupa Wattanakanjana, Chaveng Pakawatchai, Saowanit Saithong, Prapaporn Piboonphon, Ruthairat Nimthong

**Affiliations:** a Department of Chemistry, Faculty of Science, Prince of Songkla University, Hat Yai 90112, Thailand; b Department of Chemistry and Center of Excellence for Innovation in Chemistry, Faculty of Science, Prince of Songkla University, Hat Yai, Songkhla 90112, Thailand

## Abstract

In the mononuclear title complex, [CuI(C_4_H_9_N_3_S)(C_18_H_15_P)_2_], the Cu^I^ ion displays a distorted tetra­hedral coordination geometry involving two P atoms of two triphenyl­phosphane mol­ecules, one S atom of a 1-(propan-2-yl­idene)thio­semicarbazide mol­ecule and one iodide ion. In the crystal, C—H⋯π inter­actions [C—H⋯centroid distances = 3.443 (3) and 3.788 (3) Å] and N—H⋯S hydrogen bonds form layers parallel to (100). An intra­molecular N—H⋯I hydrogen bond is also observed.

## Related literature

For the potential applications of related complexes, see: Matesanz *et al.* (1999 ▶); Konstanti­nović *et al.* (2008 ▶); Zhang *et al.* (2008 ▶). For relevant examples of related discrete complexes, see: Cox *et al.* (2000 ▶); Nimthong *et al.* (2008 ▶); Pakawatchai *et al.* (2012 ▶).
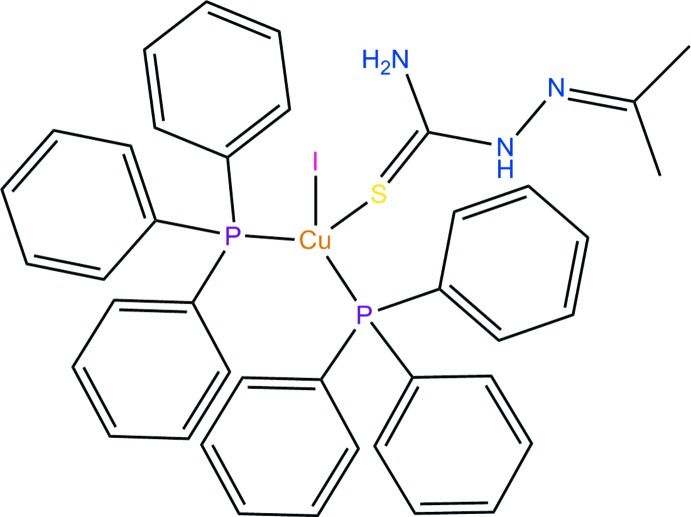


## Experimental

### 

#### Crystal data

[CuI(C_4_H_9_N_3_S)(C_18_H_15_P)_2_]
*M* *_r_* = 846.18
Triclinic, 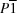

*a* = 10.8832 (6) Å
*b* = 12.5712 (7) Å
*c* = 16.0206 (8) Å
α = 98.867 (1)°
β = 100.517 (1)°
γ = 114.056 (1)°
*V* = 1903.04 (18) Å^3^
*Z* = 2
Mo *K*α radiation
μ = 1.56 mm^−1^
*T* = 293 K
0.26 × 0.21 × 0.04 mm

#### Data collection

Bruker SMART CCD area-detector diffractometer
Absorption correction: multi-scan (*SADABS*; Bruker, 2003 ▶) *T*_min_ = 0.682, *T*_max_ = 0.940
26251 measured reflections
9206 independent reflections
7690 reflections with *I* > 2σ(*I*)
*R*_int_ = 0.033

#### Refinement

*R*[*F* ^2^ > 2σ(*F* ^2^)] = 0.036
*wR*(*F* ^2^) = 0.084
*S* = 1.02
9206 reflections
444 parameters
H atoms treated by a mixture of independent and constrained refinement
Δρ_max_ = 0.66 e Å^−3^
Δρ_min_ = −0.27 e Å^−3^

### 

Data collection: *SMART* (Bruker, 1998 ▶); cell refinement: *SAINT* (Bruker, 2003 ▶); data reduction: *SAINT*; program(s) used to solve structure: *SHELXS97* (Sheldrick, 2008 ▶); program(s) used to refine structure: *SHELXL97* (Sheldrick, 2008 ▶); molecular graphics: *Mercury* (Macrae *et al.*, 2008 ▶); software used to prepare material for publication: *SHELXL97* and *publCIF* (Westrip, 2010 ▶).

## Supplementary Material

Crystal structure: contains datablock(s) I, global. DOI: 10.1107/S1600536812044066/zb2025sup1.cif

Structure factors: contains datablock(s) I. DOI: 10.1107/S1600536812044066/zb2025Isup2.hkl

Additional supplementary materials:  crystallographic information; 3D view; checkCIF report

## Figures and Tables

**Table 1:** Hydrogen-bond geometry (Å, °)

*D*—H⋯*A*	*D*—H	H⋯*A*	*D*⋯*A*	*D*—H⋯*A*
N3—H3*A*⋯S1^i^	0.85 (3)	2.62 (3)	3.447 (3)	166 (3)
N1—H1⋯I1	0.80 (3)	2.93 (3)	3.723 (2)	173 (3)

Symmetry code: (i) 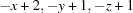.

**Fig. 1.**
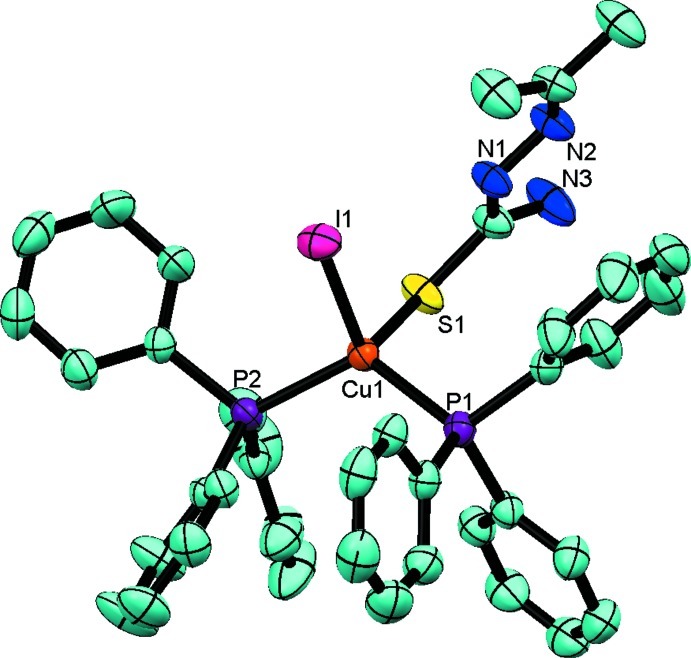
The molecular structure with displacement ellipsoids drawn at the 50% probability level. H atoms are omitted for clarity.

**Fig. 2.**
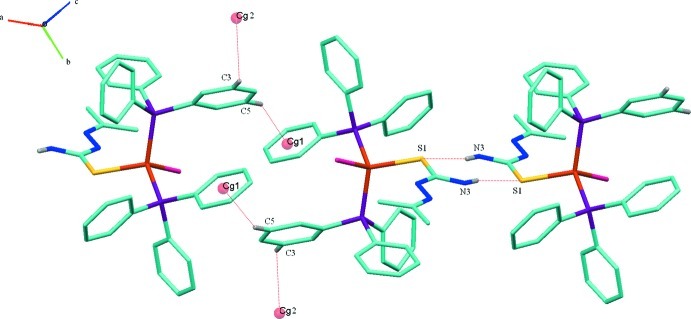
Part of the crystal structure with N—H···S hydrogen bonds and C—H···centroid interactions are linked into one dimensional chain shown as dashed lines.

## supplementary crystallographic information

### Comment

Thiosemicarbazones and thiosemicarbazone derivatives as well as their complexes have been receiving more attention, because of their potentially beneficial biochemical properties, such as antimicrobial activity (Konstantinović *et al.*, 2008), anticancer activity on cisplatin-resistant neuroblastoma cells (Zhang *et al.*, 2008), important antitumor properties and DNA binding (Matesanz *et al.*, 1999).

The molecular structure of the title compound displays the distorted tetrahedral coordination of the Cu^I^ center. (Fig. 1) The arrangement is considerably distorted since the phosphorous angle at metal site, P2—Cu1—P1 with a value of 120.68 (2)°, is much larger than the tetrahedral value 109.5°, This higher angle is counterbalanced by the bond angles of P1—Cu1—I1, P2—Cu1—I1, P1—Cu1—S1 and P2—Cu1—S1 whose values are 104.358 (19)°, 106.92 (2)°, 109.32 (3)° and 104.67 (2)°, respectively. The tetrahedral distortion is due to steric imposition of the bulky of phosphane ligands and was observed previously in analogous complex. For instance, the P—Cu—P angles of 118.63 (5)° in [CuI(C_7_H_8_N_2_S)(C_18_H_15_P)_2_]. (Nimthong *et al.*, 2008). The two Cu1—P1 and Cu1—P2 bond distances of 2.2910 (7) Å and 2.2814 (6) Å are comparable to these in CuI(C_6_H_8_N_2_S)(C_18_H_15_P)_2_ (2.2897 (5)–2.3047 (5) Å) (Pakawatchai *et al.*, 2012). The Cu1—S1 bond distance of 2.3866 (7) Å, which is larger than the value observed in tetrahedrally coordinated copper(I) halide complexes with S donors such as [CuBr(dppe)(py_2_SH)]_2_ with Cu—S bond distance of 2.3456 (13) Å (Cox *et al.*, 2000). The non-bonding distance in the molecule, N1—H1···I1, can be accepted as an intramolecular hydrogen bond with the geometry N1···I1 = 3.723 (2) Å. In the crystal packing, the C5(*sp^2^*)—H5···*π* interactions [H5···*Cg*1 = 2.948 (3) Å, C5(*sp^2^*)—H5···*Cg*1 = 3.788 (3) Å and C5(*sp^2^*)—H5···*Cg*1 = 134.62 (8)°, *Cg*1 = C19—C20—C21—C22—C23—C24 ring] and N3—H3*A*···S1 hydrogen bonds can be linked each molecule forming one dimensional chain. In addition, chains are connected through C3(*sp^2^*)—H3···*π* interactions [H3···*Cg*2 = 2.870 (3) Å, C3(*sp^2^*)—H3···*Cg*2 = 3.443 (3) Å and C3(*sp^2^*)—H3···*Cg*2 = 138.54 (7)°, *Cg*2 = C7—C8—C9—C10—C11—C12 ring] forming the two-dimensional layer networks (see Table 1 and Fig. 2).

### Experimental

Triphenylphosphane (0.28 g, 1.07 mmol) was dissolved in 30 cm^3^ of acetone at 338 K and then CuI (0.10 g, 0.53 mmol) was added. The mixture was stirred for 2 h and then thiosemicarbazide (0.05 g, 0.55 mmol) was added and the new reaction mixture was heated under reflux for 5 h where upon the precipitate gradually disappeared. The resulting clear solution was filtered off and left to evaporate at room temperature. The crystalline complex, which was deposited upon standing for several days, was filtered off and dried in *vacuo*.

### Refinement

The H atoms were positioned geometrically and refined using a riding model, with C—H = 0.93 with *U*_iso_(H) = 1.2 *U*_eq_(C) and 0.96 Å with *U*_iso_(H) = 1.5 *U*_eq_(C) for for H atoms on C(*sp^2^*) and C(*sp^3^*), respectively. All H atoms bonded to N atoms were located in a difference Fourier map and refined isotropically.

### Crystal data

**Table:**

[CuI(C_4_H_9_N_3_S)(C_18_H_15_P)_2_]	*Z* = 2
*M**_r_* = 846.18	*F*(000) = 856
Triclinic, *P*1	*D*_x_ = 1.477 Mg m^−^^3^
Hall symbol: -P 1	Mo *K*α radiation, λ = 0.71073 Å
*a* = 10.8832 (6) Å	Cell parameters from 7509 reflections
*b* = 12.5712 (7) Å	θ = 2.2–26.4°
*c* = 16.0206 (8) Å	µ = 1.56 mm^−^^1^
α = 98.867 (1)°	*T* = 293 K
β = 100.517 (1)°	Plate, colourless
γ = 114.056 (1)°	0.26 × 0.21 × 0.04 mm
*V* = 1903.04 (18) Å^3^	

### Data collection

**Table:**

Bruker SMART CCD area-detector diffractometer	9206 independent reflections
Radiation source: fine-focus sealed tube	7690 reflections with *I* > 2σ(*I*)
Graphite monochromator	*R*_int_ = 0.033
Frames, each covering 0.3 ° in ω scans	θ_max_ = 28.1°, θ_min_ = 1.3°
Absorption correction: multi-scan (*SADABS*; Bruker, 2003)	*h* = −14→14
*T*_min_ = 0.682, *T*_max_ = 0.940	*k* = −16→16
26251 measured reflections	*l* = −21→21

### Refinement

**Table:**

Refinement on *F*^2^	Primary atom site location: structure-invariant direct methods
Least-squares matrix: full	Secondary atom site location: difference Fourier map
*R*[*F*^2^ > 2σ(*F*^2^)] = 0.036	Hydrogen site location: inferred from neighbouring sites
*wR*(*F*^2^) = 0.084	H atoms treated by a mixture of independent and constrained refinement
*S* = 1.02	*w* = 1/[σ^2^(*F*_o_^2^) + (0.0373*P*)^2^ + 0.6367*P*] where *P* = (*F*_o_^2^ + 2*F*_c_^2^)/3
9206 reflections	(Δ/σ)_max_ = 0.002
444 parameters	Δρ_max_ = 0.66 e Å^−^^3^
0 restraints	Δρ_min_ = −0.27 e Å^−^^3^

### Special details

**Table:**

Geometry. All e.s.d.'s (except the e.s.d. in the dihedral angle between two l.s. planes) are estimated using the full covariance matrix. The cell e.s.d.'s are taken into account individually in the estimation of e.s.d.'s in distances, angles and torsion angles; correlations between e.s.d.'s in cell parameters are only used when they are defined by crystal symmetry. An approximate (isotropic) treatment of cell e.s.d.'s is used for estimating e.s.d.'s involving l.s. planes.
Refinement. Refinement of *F*^2^ against ALL reflections. The weighted *R*-factor *wR* and goodness of fit *S* are based on *F*^2^, conventional *R*-factors *R* are based on *F*, with *F* set to zero for negative *F*^2^. The threshold expression of *F*^2^ > σ(*F*^2^) is used only for calculating *R*-factors(gt) *etc*. and is not relevant to the choice of reflections for refinement. *R*-factors based on *F*^2^ are statistically about twice as large as those based on *F*, and *R*- factors based on ALL data will be even larger.

### Fractional atomic coordinates and isotropic or equivalent isotropic displacement parameters (Å^2^)

**Table:**

	*x*	*y*	*z*	*U*_iso_*/*U*_eq_	
C1	0.9764 (3)	0.3109 (2)	0.05375 (16)	0.0384 (5)	
C2	0.8564 (3)	0.2797 (3)	−0.01226 (18)	0.0496 (7)	
H2	0.7874	0.2998	0.0006	0.060*	
C3	0.8377 (4)	0.2189 (3)	−0.0971 (2)	0.0630 (8)	
H3	0.7560	0.1977	−0.1402	0.076*	
C4	0.9382 (4)	0.1899 (3)	−0.1178 (2)	0.0627 (9)	
H4	0.9261	0.1508	−0.1751	0.075*	
C5	1.0567 (4)	0.2185 (3)	−0.05395 (19)	0.0544 (7)	
H5	1.1252	0.1987	−0.0680	0.065*	
C6	1.0759 (3)	0.2770 (2)	0.03164 (17)	0.0435 (6)	
H6	1.1559	0.2939	0.0747	0.052*	
C7	1.1523 (3)	0.5247 (2)	0.20007 (17)	0.0402 (6)	
C8	1.2636 (3)	0.5503 (3)	0.1635 (2)	0.0606 (8)	
H8	1.2571	0.4948	0.1151	0.073*	
C9	1.3843 (3)	0.6576 (3)	0.1979 (3)	0.0781 (11)	
H9	1.4586	0.6733	0.1730	0.094*	
C10	1.3953 (4)	0.7406 (3)	0.2683 (3)	0.0765 (11)	
H10	1.4773	0.8121	0.2918	0.092*	
C11	1.2856 (4)	0.7183 (3)	0.3039 (2)	0.0780 (11)	
H11	1.2919	0.7758	0.3509	0.094*	
C12	1.1647 (4)	0.6105 (3)	0.2707 (2)	0.0606 (8)	
H12	1.0910	0.5957	0.2961	0.073*	
C13	0.8550 (3)	0.4181 (2)	0.16390 (16)	0.0371 (5)	
C14	0.7594 (3)	0.3687 (2)	0.21055 (18)	0.0435 (6)	
H14	0.7732	0.3200	0.2457	0.052*	
C15	0.6439 (3)	0.3907 (3)	0.2057 (2)	0.0526 (7)	
H15	0.5804	0.3565	0.2371	0.063*	
C16	0.6236 (3)	0.4628 (3)	0.1546 (2)	0.0594 (8)	
H16	0.5453	0.4767	0.1505	0.071*	
C17	0.7184 (3)	0.5151 (3)	0.1092 (2)	0.0590 (8)	
H17	0.7041	0.5645	0.0749	0.071*	
C18	0.8342 (3)	0.4947 (2)	0.11416 (19)	0.0487 (6)	
H18	0.8989	0.5319	0.0844	0.058*	
C19	0.7536 (2)	−0.0101 (2)	0.13076 (15)	0.0360 (5)	
C20	0.6175 (3)	−0.1014 (3)	0.10113 (19)	0.0613 (9)	
H20	0.5629	−0.1203	0.1401	0.074*	
C21	0.5629 (3)	−0.1644 (3)	0.0135 (2)	0.0762 (11)	
H21	0.4716	−0.2255	−0.0060	0.091*	
C22	0.6418 (3)	−0.1376 (3)	−0.04459 (19)	0.0640 (9)	
H22	0.6039	−0.1794	−0.1035	0.077*	
C23	0.7762 (3)	−0.0493 (3)	−0.01590 (18)	0.0511 (7)	
H23	0.8309	−0.0322	−0.0550	0.061*	
C24	0.8314 (3)	0.0147 (2)	0.07120 (16)	0.0404 (6)	
H24	0.9227	0.0758	0.0899	0.048*	
C25	0.7027 (3)	0.0710 (2)	0.29446 (17)	0.0377 (5)	
C26	0.7158 (3)	0.0687 (3)	0.38138 (19)	0.0557 (7)	
H26	0.7923	0.0628	0.4131	0.067*	
C27	0.6157 (5)	0.0750 (3)	0.4216 (3)	0.0819 (12)	
H27	0.6256	0.0734	0.4803	0.098*	
C28	0.5032 (5)	0.0836 (4)	0.3762 (4)	0.0937 (15)	
H28	0.4363	0.0875	0.4035	0.112*	
C29	0.4888 (4)	0.0864 (4)	0.2902 (3)	0.0862 (13)	
H29	0.4114	0.0915	0.2589	0.103*	
C30	0.5887 (3)	0.0819 (3)	0.2494 (2)	0.0592 (8)	
H30	0.5793	0.0861	0.1913	0.071*	
C31	0.8974 (2)	−0.0266 (2)	0.29335 (15)	0.0340 (5)	
C32	1.0122 (3)	0.0236 (3)	0.3647 (2)	0.0566 (8)	
H32	1.0604	0.1069	0.3858	0.068*	
C33	1.0572 (4)	−0.0485 (3)	0.4057 (2)	0.0738 (10)	
H33	1.1339	−0.0134	0.4549	0.089*	
C34	0.9894 (4)	−0.1706 (3)	0.3743 (2)	0.0640 (9)	
H34	1.0204	−0.2188	0.4015	0.077*	
C35	0.8769 (4)	−0.2213 (3)	0.3035 (2)	0.0599 (8)	
H35	0.8305	−0.3047	0.2823	0.072*	
C36	0.8299 (3)	−0.1508 (2)	0.26227 (18)	0.0478 (6)	
H36	0.7527	−0.1870	0.2134	0.057*	
C37	1.1977 (3)	0.4956 (2)	0.45169 (16)	0.0399 (5)	
C38	1.5399 (3)	0.6307 (2)	0.43728 (17)	0.0436 (6)	
C39	1.5519 (3)	0.5446 (3)	0.3685 (2)	0.0624 (8)	
H39A	1.5303	0.4699	0.3847	0.094*	
H39B	1.6455	0.5777	0.3627	0.094*	
H39C	1.4875	0.5304	0.3135	0.094*	
C40	1.6672 (3)	0.7473 (3)	0.4791 (2)	0.0663 (9)	
H40A	1.6877	0.7931	0.4363	0.099*	
H40B	1.7449	0.7321	0.5013	0.099*	
H40C	1.6511	0.7921	0.5265	0.099*	
N1	1.3115 (2)	0.5081 (2)	0.42585 (14)	0.0410 (5)	
N2	1.4325 (2)	0.6151 (2)	0.46382 (14)	0.0467 (5)	
N3	1.2117 (3)	0.5846 (2)	0.51463 (18)	0.0631 (8)	
P1	0.99834 (6)	0.37784 (5)	0.16846 (4)	0.03298 (13)	
P2	0.84194 (6)	0.07508 (5)	0.24472 (4)	0.03094 (13)	
S1	1.04319 (6)	0.36891 (6)	0.40876 (4)	0.04410 (16)	
Cu1	1.01628 (3)	0.26360 (2)	0.264156 (18)	0.03273 (8)	
I1	1.243129 (17)	0.239385 (16)	0.254284 (11)	0.04628 (7)	
H1	1.304 (3)	0.454 (3)	0.388 (2)	0.056*	
H3A	1.140 (3)	0.583 (3)	0.529 (2)	0.056*	
H3B	1.290 (3)	0.643 (3)	0.535 (2)	0.056*	

### Atomic displacement parameters (Å^2^)

**Table:**

	*U*^11^	*U*^22^	*U*^33^	*U*^12^	*U*^13^	*U*^23^
C1	0.0453 (14)	0.0323 (12)	0.0416 (13)	0.0173 (11)	0.0158 (11)	0.0157 (10)
C2	0.0542 (16)	0.0495 (16)	0.0475 (15)	0.0279 (14)	0.0099 (13)	0.0100 (13)
C3	0.077 (2)	0.065 (2)	0.0428 (16)	0.0356 (18)	0.0025 (15)	0.0104 (14)
C4	0.099 (3)	0.0581 (19)	0.0399 (16)	0.0413 (19)	0.0238 (17)	0.0133 (14)
C5	0.081 (2)	0.0505 (17)	0.0540 (17)	0.0397 (16)	0.0368 (16)	0.0217 (14)
C6	0.0530 (15)	0.0426 (14)	0.0453 (14)	0.0258 (13)	0.0202 (12)	0.0184 (12)
C7	0.0381 (13)	0.0318 (12)	0.0475 (14)	0.0119 (11)	0.0078 (11)	0.0167 (11)
C8	0.0480 (16)	0.0408 (16)	0.095 (2)	0.0169 (13)	0.0287 (17)	0.0216 (16)
C9	0.0428 (17)	0.052 (2)	0.136 (4)	0.0129 (15)	0.027 (2)	0.035 (2)
C10	0.055 (2)	0.0445 (19)	0.093 (3)	−0.0013 (15)	−0.0123 (19)	0.0233 (19)
C11	0.095 (3)	0.0460 (19)	0.0530 (19)	0.0045 (18)	0.0016 (19)	0.0034 (15)
C12	0.068 (2)	0.0408 (16)	0.0518 (17)	0.0064 (14)	0.0162 (15)	0.0069 (13)
C13	0.0378 (13)	0.0306 (12)	0.0417 (13)	0.0153 (10)	0.0112 (10)	0.0059 (10)
C14	0.0414 (14)	0.0405 (14)	0.0468 (15)	0.0174 (12)	0.0136 (12)	0.0067 (12)
C15	0.0418 (15)	0.0529 (17)	0.0617 (18)	0.0217 (13)	0.0204 (13)	0.0019 (14)
C16	0.0450 (16)	0.0563 (18)	0.073 (2)	0.0301 (15)	0.0085 (15)	−0.0055 (16)
C17	0.0617 (19)	0.0519 (18)	0.071 (2)	0.0360 (16)	0.0111 (16)	0.0134 (15)
C18	0.0525 (16)	0.0418 (15)	0.0600 (17)	0.0255 (13)	0.0207 (14)	0.0167 (13)
C19	0.0371 (12)	0.0321 (12)	0.0308 (12)	0.0103 (10)	0.0050 (10)	0.0060 (10)
C20	0.0433 (16)	0.069 (2)	0.0416 (15)	0.0014 (14)	0.0107 (12)	−0.0001 (14)
C21	0.0442 (17)	0.085 (3)	0.0500 (18)	−0.0024 (16)	0.0006 (14)	−0.0140 (17)
C22	0.064 (2)	0.071 (2)	0.0333 (14)	0.0187 (17)	0.0003 (14)	−0.0025 (14)
C23	0.0654 (18)	0.0476 (16)	0.0367 (14)	0.0218 (14)	0.0157 (13)	0.0088 (12)
C24	0.0442 (14)	0.0317 (12)	0.0387 (13)	0.0120 (11)	0.0105 (11)	0.0066 (10)
C25	0.0365 (12)	0.0275 (12)	0.0487 (14)	0.0112 (10)	0.0176 (11)	0.0105 (10)
C26	0.0610 (18)	0.0609 (19)	0.0456 (16)	0.0255 (15)	0.0230 (14)	0.0098 (14)
C27	0.098 (3)	0.080 (3)	0.076 (2)	0.035 (2)	0.057 (2)	0.014 (2)
C28	0.094 (3)	0.074 (3)	0.147 (4)	0.048 (2)	0.086 (3)	0.027 (3)
C29	0.067 (2)	0.082 (3)	0.154 (4)	0.055 (2)	0.060 (3)	0.056 (3)
C30	0.0519 (17)	0.0611 (19)	0.078 (2)	0.0312 (15)	0.0245 (16)	0.0308 (17)
C31	0.0380 (12)	0.0302 (12)	0.0339 (12)	0.0143 (10)	0.0117 (10)	0.0094 (9)
C32	0.0561 (17)	0.0380 (15)	0.0565 (17)	0.0128 (13)	−0.0086 (14)	0.0119 (13)
C33	0.068 (2)	0.062 (2)	0.074 (2)	0.0213 (17)	−0.0143 (17)	0.0292 (18)
C34	0.072 (2)	0.064 (2)	0.076 (2)	0.0413 (18)	0.0230 (18)	0.0379 (18)
C35	0.079 (2)	0.0348 (15)	0.067 (2)	0.0246 (15)	0.0236 (17)	0.0146 (14)
C36	0.0530 (16)	0.0369 (14)	0.0439 (15)	0.0144 (12)	0.0090 (12)	0.0050 (11)
C37	0.0336 (12)	0.0422 (14)	0.0366 (13)	0.0154 (11)	0.0074 (10)	−0.0022 (11)
C38	0.0346 (13)	0.0481 (15)	0.0378 (13)	0.0128 (11)	0.0067 (10)	0.0030 (11)
C39	0.0486 (16)	0.062 (2)	0.065 (2)	0.0165 (15)	0.0251 (15)	−0.0037 (16)
C40	0.0389 (15)	0.059 (2)	0.070 (2)	0.0033 (14)	0.0133 (15)	−0.0095 (16)
N1	0.0322 (10)	0.0391 (12)	0.0392 (12)	0.0109 (9)	0.0082 (9)	−0.0073 (9)
N2	0.0338 (11)	0.0459 (13)	0.0429 (12)	0.0089 (10)	0.0072 (9)	−0.0067 (10)
N3	0.0371 (13)	0.0562 (16)	0.0702 (18)	0.0093 (12)	0.0176 (13)	−0.0246 (13)
P1	0.0333 (3)	0.0291 (3)	0.0381 (3)	0.0134 (2)	0.0130 (3)	0.0103 (2)
P2	0.0305 (3)	0.0283 (3)	0.0281 (3)	0.0090 (2)	0.0061 (2)	0.0057 (2)
S1	0.0330 (3)	0.0448 (4)	0.0415 (3)	0.0101 (3)	0.0127 (3)	−0.0064 (3)
Cu1	0.03092 (14)	0.02914 (15)	0.03466 (15)	0.01103 (12)	0.00811 (11)	0.00656 (12)
I1	0.03963 (10)	0.05671 (12)	0.04491 (11)	0.02808 (9)	0.01026 (7)	0.00238 (8)

### Geometric parameters (Å, º)

**Table:**

C1—C2	1.389 (4)	C23—H23	0.9300
C1—C6	1.395 (4)	C24—H24	0.9300
C1—P1	1.828 (3)	C25—C26	1.380 (4)
C2—C3	1.387 (4)	C25—C30	1.385 (4)
C2—H2	0.9300	C25—P2	1.824 (2)
C3—C4	1.364 (5)	C26—C27	1.386 (4)
C3—H3	0.9300	C26—H26	0.9300
C4—C5	1.366 (5)	C27—C28	1.359 (6)
C4—H4	0.9300	C27—H27	0.9300
C5—C6	1.387 (4)	C28—C29	1.365 (6)
C5—H5	0.9300	C28—H28	0.9300
C6—H6	0.9300	C29—C30	1.382 (5)
C7—C12	1.383 (4)	C29—H29	0.9300
C7—C8	1.383 (4)	C30—H30	0.9300
C7—P1	1.831 (2)	C31—C32	1.370 (4)
C8—C9	1.382 (4)	C31—C36	1.384 (3)
C8—H8	0.9300	C31—P2	1.836 (2)
C9—C10	1.364 (6)	C32—C33	1.387 (4)
C9—H9	0.9300	C32—H32	0.9300
C10—C11	1.362 (5)	C33—C34	1.362 (5)
C10—H10	0.9300	C33—H33	0.9300
C11—C12	1.386 (4)	C34—C35	1.351 (4)
C11—H11	0.9300	C34—H34	0.9300
C12—H12	0.9300	C35—C36	1.382 (4)
C13—C14	1.388 (3)	C35—H35	0.9300
C13—C18	1.401 (4)	C36—H36	0.9300
C13—P1	1.819 (3)	C37—N3	1.323 (3)
C14—C15	1.384 (4)	C37—N1	1.334 (3)
C14—H14	0.9300	C37—S1	1.701 (3)
C15—C16	1.367 (4)	C38—N2	1.267 (3)
C15—H15	0.9300	C38—C39	1.484 (4)
C16—C17	1.377 (5)	C38—C40	1.490 (4)
C16—H16	0.9300	C39—H39A	0.9600
C17—C18	1.376 (4)	C39—H39B	0.9600
C17—H17	0.9300	C39—H39C	0.9600
C18—H18	0.9300	C40—H40A	0.9600
C19—C24	1.377 (3)	C40—H40B	0.9600
C19—C20	1.388 (4)	C40—H40C	0.9600
C19—P2	1.823 (2)	N1—N2	1.388 (3)
C20—C21	1.385 (4)	N1—H1	0.80 (3)
C20—H20	0.9300	N3—H3A	0.85 (3)
C21—C22	1.368 (4)	N3—H3B	0.83 (3)
C21—H21	0.9300	P1—Cu1	2.2910 (7)
C22—C23	1.362 (4)	P2—Cu1	2.2814 (6)
C22—H22	0.9300	S1—Cu1	2.3866 (7)
C23—C24	1.382 (4)	Cu1—I1	2.6369 (3)
			
C2—C1—C6	117.4 (2)	C25—C26—H26	119.8
C2—C1—P1	122.6 (2)	C27—C26—H26	119.8
C6—C1—P1	119.7 (2)	C28—C27—C26	120.5 (4)
C3—C2—C1	121.0 (3)	C28—C27—H27	119.7
C3—C2—H2	119.5	C26—C27—H27	119.7
C1—C2—H2	119.5	C27—C28—C29	119.8 (3)
C4—C3—C2	120.6 (3)	C27—C28—H28	120.1
C4—C3—H3	119.7	C29—C28—H28	120.1
C2—C3—H3	119.7	C28—C29—C30	120.4 (4)
C3—C4—C5	119.6 (3)	C28—C29—H29	119.8
C3—C4—H4	120.2	C30—C29—H29	119.8
C5—C4—H4	120.2	C29—C30—C25	120.5 (3)
C4—C5—C6	120.7 (3)	C29—C30—H30	119.8
C4—C5—H5	119.7	C25—C30—H30	119.8
C6—C5—H5	119.7	C32—C31—C36	118.3 (2)
C5—C6—C1	120.7 (3)	C32—C31—P2	117.90 (19)
C5—C6—H6	119.6	C36—C31—P2	123.84 (19)
C1—C6—H6	119.6	C31—C32—C33	120.7 (3)
C12—C7—C8	118.0 (3)	C31—C32—H32	119.7
C12—C7—P1	118.0 (2)	C33—C32—H32	119.7
C8—C7—P1	123.7 (2)	C34—C33—C32	120.3 (3)
C9—C8—C7	120.7 (3)	C34—C33—H33	119.9
C9—C8—H8	119.7	C32—C33—H33	119.9
C7—C8—H8	119.7	C35—C34—C33	119.7 (3)
C10—C9—C8	120.5 (3)	C35—C34—H34	120.1
C10—C9—H9	119.7	C33—C34—H34	120.1
C8—C9—H9	119.7	C34—C35—C36	120.8 (3)
C11—C10—C9	119.7 (3)	C34—C35—H35	119.6
C11—C10—H10	120.2	C36—C35—H35	119.6
C9—C10—H10	120.2	C35—C36—C31	120.3 (3)
C10—C11—C12	120.4 (4)	C35—C36—H36	119.8
C10—C11—H11	119.8	C31—C36—H36	119.8
C12—C11—H11	119.8	N3—C37—N1	116.9 (2)
C7—C12—C11	120.7 (3)	N3—C37—S1	121.4 (2)
C7—C12—H12	119.6	N1—C37—S1	121.72 (19)
C11—C12—H12	119.6	N2—C38—C39	126.6 (2)
C14—C13—C18	118.1 (2)	N2—C38—C40	116.9 (2)
C14—C13—P1	118.57 (19)	C39—C38—C40	116.5 (2)
C18—C13—P1	123.3 (2)	C38—C39—H39A	109.5
C15—C14—C13	121.2 (3)	C38—C39—H39B	109.5
C15—C14—H14	119.4	H39A—C39—H39B	109.5
C13—C14—H14	119.4	C38—C39—H39C	109.5
C16—C15—C14	119.6 (3)	H39A—C39—H39C	109.5
C16—C15—H15	120.2	H39B—C39—H39C	109.5
C14—C15—H15	120.2	C38—C40—H40A	109.5
C15—C16—C17	120.4 (3)	C38—C40—H40B	109.5
C15—C16—H16	119.8	H40A—C40—H40B	109.5
C17—C16—H16	119.8	C38—C40—H40C	109.5
C18—C17—C16	120.5 (3)	H40A—C40—H40C	109.5
C18—C17—H17	119.8	H40B—C40—H40C	109.5
C16—C17—H17	119.8	C37—N1—N2	117.7 (2)
C17—C18—C13	120.2 (3)	C37—N1—H1	117 (2)
C17—C18—H18	119.9	N2—N1—H1	125 (2)
C13—C18—H18	119.9	C38—N2—N1	118.6 (2)
C24—C19—C20	118.3 (2)	C37—N3—H3A	119 (2)
C24—C19—P2	116.79 (18)	C37—N3—H3B	118 (2)
C20—C19—P2	124.8 (2)	H3A—N3—H3B	123 (3)
C21—C20—C19	120.0 (3)	C13—P1—C1	103.89 (11)
C21—C20—H20	120.0	C13—P1—C7	102.85 (11)
C19—C20—H20	120.0	C1—P1—C7	106.61 (12)
C22—C21—C20	120.7 (3)	C13—P1—Cu1	115.50 (8)
C22—C21—H21	119.7	C1—P1—Cu1	115.50 (8)
C20—C21—H21	119.7	C7—P1—Cu1	111.31 (8)
C23—C22—C21	119.8 (3)	C19—P2—C25	105.23 (12)
C23—C22—H22	120.1	C19—P2—C31	103.20 (11)
C21—C22—H22	120.1	C25—P2—C31	102.33 (11)
C22—C23—C24	120.0 (3)	C19—P2—Cu1	115.17 (8)
C22—C23—H23	120.0	C25—P2—Cu1	114.47 (8)
C24—C23—H23	120.0	C31—P2—Cu1	114.92 (8)
C19—C24—C23	121.2 (2)	C37—S1—Cu1	113.83 (9)
C19—C24—H24	119.4	P2—Cu1—P1	120.68 (2)
C23—C24—H24	119.4	P2—Cu1—S1	104.67 (2)
C26—C25—C30	118.3 (3)	P1—Cu1—S1	109.32 (3)
C26—C25—P2	120.0 (2)	P2—Cu1—I1	106.92 (2)
C30—C25—P2	121.4 (2)	P1—Cu1—I1	104.358 (19)
C25—C26—C27	120.4 (3)	S1—Cu1—I1	110.821 (19)
			
C6—C1—C2—C3	−1.0 (4)	C18—C13—P1—C7	53.7 (2)
P1—C1—C2—C3	−173.9 (2)	C14—C13—P1—Cu1	−6.2 (2)
C1—C2—C3—C4	−0.9 (5)	C18—C13—P1—Cu1	175.13 (19)
C2—C3—C4—C5	1.5 (5)	C2—C1—P1—C13	−11.9 (2)
C3—C4—C5—C6	−0.1 (5)	C6—C1—P1—C13	175.3 (2)
C4—C5—C6—C1	−1.9 (4)	C2—C1—P1—C7	−120.1 (2)
C2—C1—C6—C5	2.4 (4)	C6—C1—P1—C7	67.1 (2)
P1—C1—C6—C5	175.5 (2)	C2—C1—P1—Cu1	115.6 (2)
C12—C7—C8—C9	1.3 (5)	C6—C1—P1—Cu1	−57.2 (2)
P1—C7—C8—C9	−171.6 (3)	C12—C7—P1—C13	48.9 (2)
C7—C8—C9—C10	−0.6 (5)	C8—C7—P1—C13	−138.1 (2)
C8—C9—C10—C11	−1.0 (6)	C12—C7—P1—C1	157.9 (2)
C9—C10—C11—C12	1.8 (6)	C8—C7—P1—C1	−29.1 (3)
C8—C7—C12—C11	−0.6 (5)	C12—C7—P1—Cu1	−75.3 (2)
P1—C7—C12—C11	172.8 (3)	C8—C7—P1—Cu1	97.6 (2)
C10—C11—C12—C7	−1.0 (5)	C24—C19—P2—C25	156.56 (19)
C18—C13—C14—C15	2.4 (4)	C20—C19—P2—C25	−26.9 (3)
P1—C13—C14—C15	−176.4 (2)	C24—C19—P2—C31	−96.5 (2)
C13—C14—C15—C16	−0.4 (4)	C20—C19—P2—C31	80.0 (3)
C14—C15—C16—C17	−1.0 (4)	C24—C19—P2—Cu1	29.5 (2)
C15—C16—C17—C18	0.5 (5)	C20—C19—P2—Cu1	−154.0 (2)
C16—C17—C18—C13	1.5 (5)	C26—C25—P2—C19	146.5 (2)
C14—C13—C18—C17	−2.9 (4)	C30—C25—P2—C19	−39.7 (2)
P1—C13—C18—C17	175.7 (2)	C26—C25—P2—C31	39.0 (2)
C24—C19—C20—C21	−0.4 (5)	C30—C25—P2—C31	−147.3 (2)
P2—C19—C20—C21	−176.8 (3)	C26—C25—P2—Cu1	−86.0 (2)
C19—C20—C21—C22	−0.1 (6)	C30—C25—P2—Cu1	87.8 (2)
C20—C21—C22—C23	1.0 (6)	C32—C31—P2—C19	153.3 (2)
C21—C22—C23—C24	−1.6 (5)	C36—C31—P2—C19	−26.8 (2)
C20—C19—C24—C23	−0.2 (4)	C32—C31—P2—C25	−97.5 (2)
P2—C19—C24—C23	176.6 (2)	C36—C31—P2—C25	82.4 (2)
C22—C23—C24—C19	1.1 (4)	C32—C31—P2—Cu1	27.1 (2)
C30—C25—C26—C27	1.0 (4)	C36—C31—P2—Cu1	−153.0 (2)
P2—C25—C26—C27	175.0 (3)	N3—C37—S1—Cu1	156.4 (2)
C25—C26—C27—C28	0.0 (6)	N1—C37—S1—Cu1	−26.1 (3)
C26—C27—C28—C29	−0.3 (6)	C19—P2—Cu1—P1	35.40 (10)
C27—C28—C29—C30	−0.6 (6)	C25—P2—Cu1—P1	−86.79 (10)
C28—C29—C30—C25	1.7 (6)	C31—P2—Cu1—P1	155.17 (8)
C26—C25—C30—C29	−1.9 (5)	C19—P2—Cu1—S1	158.97 (9)
P2—C25—C30—C29	−175.8 (3)	C25—P2—Cu1—S1	36.78 (10)
C36—C31—C32—C33	−1.5 (5)	C31—P2—Cu1—S1	−81.26 (9)
P2—C31—C32—C33	178.4 (3)	C19—P2—Cu1—I1	−83.40 (9)
C31—C32—C33—C34	1.4 (6)	C25—P2—Cu1—I1	154.41 (9)
C32—C33—C34—C35	−0.8 (6)	C31—P2—Cu1—I1	36.36 (9)
C33—C34—C35—C36	0.3 (5)	C13—P1—Cu1—P2	63.00 (9)
C34—C35—C36—C31	−0.4 (5)	C1—P1—Cu1—P2	−58.48 (10)
C32—C31—C36—C35	1.0 (4)	C7—P1—Cu1—P2	179.78 (10)
P2—C31—C36—C35	−178.9 (2)	C13—P1—Cu1—S1	−58.33 (9)
N3—C37—N1—N2	−3.0 (4)	C1—P1—Cu1—S1	−179.81 (9)
S1—C37—N1—N2	179.31 (19)	C7—P1—Cu1—S1	58.45 (10)
C39—C38—N2—N1	−0.5 (5)	C13—P1—Cu1—I1	−176.92 (9)
C40—C38—N2—N1	179.4 (3)	C1—P1—Cu1—I1	61.60 (9)
C37—N1—N2—C38	−176.9 (3)	C7—P1—Cu1—I1	−60.14 (10)
C14—C13—P1—C1	121.3 (2)	C37—S1—Cu1—P2	161.89 (10)
C18—C13—P1—C1	−57.3 (2)	C37—S1—Cu1—P1	−67.52 (11)
C14—C13—P1—C7	−127.7 (2)	C37—S1—Cu1—I1	46.97 (11)

### Hydrogen-bond geometry (Å, º)

**Table:**

*D*—H···*A*	*D*—H	H···*A*	*D*···*A*	*D*—H···*A*
N3—H3*A*···S1^i^	0.85 (3)	2.62 (3)	3.447 (3)	166 (3)
N1—H1···I1	0.80 (3)	2.93 (3)	3.723 (2)	173 (3)

Symmetry code: (i) −*x*+2, −*y*+1, −*z*+1.
